# Association of Radiological Parameters to Functional Outcomes after Distal Radius Fracture Fixation with Volar Locking Plate: A Prospective Cohort Study

**DOI:** 10.5704/MOJ.2511.016

**Published:** 2025-11

**Authors:** RS Bilal, HR Rizwan, Z Marij, MH Pervaiz, R Haroon

**Affiliations:** Department of Orthopaedics, Aga Khan University Hospital, Karachi, Pakistan

**Keywords:** distal radius fractures, PRWE score, volar locking plate

## Abstract

**Introduction:**

Distal radius fractures are among the most common orthopaedic injuries, and volar locking plate fixation is a widely used surgical method. The restoration of radiological parameters post-operatively is believed to be associated with reliable functional recovery. This study aims to evaluate the correlation between radiological parameters and functional outcomes following distal radius fracture fixation.

**Materials and methods:**

This prospective cohort study included patients with distal radius fractures treated with volar locking plates. Radiological parameters, including volar tilt, radial inclination, radial height, and ulnar variance, were assessed post-operatively. Functional outcomes were measured using the Patient Rated Wrist Evaluation score at predefined follow-up intervals. Correlation analyses were conducted to assess the relationship between radiological restoration and clinical outcomes. Pearson correlation coefficient was applied to assess the relationship between the outcome variable and predictor variables. Simple linear regression was initially performed with a significance threshold of 25%, followed by stepwise multiple linear regression using a 5% significance level.

**Results:**

A total of 78 patients (56.4% male, 43.6% female) with a mean age of 50.3 ± 14.7 years were included. Most patients sustained partial or complete intra-articular distal radius fractures. The mean cumulative PRWE score at 3 months was 42.4 ± 6.6, with mean pain and function sub-scores of 21.24 and 21.34, respectively. The study findings showed that increased age and ulnar variance were significantly associated with worse PRWE scores while greater palmar tilt was associated with better outcomes. Radial height and radial inclination were not significantly correlated with functional scores.

**Conclusion:**

Understanding the relationship between radiological alignment and functional outcomes can aid in optimising surgical techniques and improving patient recovery.

## Introduction

Distal radius fractures account for a significant proportion of orthopaedic injuries, particularly among elderly patients with osteoporosis and younger individuals engaged in high-impact activities. Recent data from a large U.S. cohort identified the radius as the most frequently fractured long bone. Distal radius fractures contribute significantly to healthcare demand, comprising roughly 16% of emergency department cases and between 26% to 46% of skeletal fractures managed in primary care^1^. The preferred surgical treatment for unstable or intra-articular fractures is open reduction and internal fixation (ORIF) using volar locking plates depending upon the fracture configuration^2-4^. Recent advances have specified for a fragment or column specific fixation^5,6^, however volar locking plate remains the mainstay of the treatment due to its widespread availability and surgeon familiarity among the surgeons^6-8^. Despite advances in surgical techniques and implant design, variability in patient outcomes persists, however the goal of fixation remains the same that is to restore anatomical alignment, which itself is hypothesised to influence functional recovery and long-term wrist function.

Radiological parameters, such as volar tilt, radial inclination, radial height, and ulnar variance, are critical in evaluating post-operative alignment. Several studies suggest that improper restoration of these parameters can lead to poor functional outcomes, including limited wrist mobility, chronic pain, and reduced grip strength^9,10^. However, the extent to which these radiological parameters correlate with functional recovery remains a subject of debate^11^.

This prospective cohort study aims to investigate the association between post-operative radiological parameters and functional outcomes in patients who underwent Volar locking plate fixation for distal radius fractures. By analysing the relationship between objective radiographic data and validated functional scores, this study seeks to clarify which parameters are most predictive of recovery.

## Materials and Methods

It is a prospective cohort study was conducted at Aga Khan University Hospital, Karachi, Pakistan including patients diagnosed with distal radius fractures requiring surgical fixation after approval from the institutional ethical review committee (ERC#2023-0526-25942). All the adult patients (i.e 18 years or above) with closed unstable or intra-articular distal radius fractures who underwent ORIF with volar locking plate and followed at least up to three months were included in the study. While post-operative rehabilitation was routinely recommended, adherence to physiotherapy was not monitored or used as a criterion for inclusion. Patients were excluded if they presented with open distal radius fractures, had a history of wrist surgery, or suffered from localised pre-existing wrist pathologies that could interfere with post-operative functional assessment. These included prior fractures with malunion, advanced wrist osteoarthritis, or inflammatory arthropathies such as rheumatoid arthritis.

Post-operative radiographs were analysed to measure the volar tilt, radial inclination, radial height and ulnar variance. Functional assessment was measured at three months using Patient-Reported Wrist Evaluation (PRWE) score. It is a 15-question self-assessment tool used to evaluate wrist pain and its impact on daily life and comprises of two sections- pain and function. The pain section has five questions, and the function section has ten questions about specific and usual activities. Final score ranges from 0 to 100, with lower scores indicating better outcome^12^.

Data was entered on a preformed structural questionnaire and entered and analysed via Stata version 20. Continuous data like age and radiographic parameters are represented as means and standard deviation and categorical data as frequencies and percentages. Furthermore, correlation and linear regression modelling was done to assess the relationship between radiological parameters and PRWE scores keeping level of significance at 5%. Pearson’s correlation analysis was employed to explore the relationship between the outcome and predictor variables. Subsequently, simple linear regression was conducted using a liberal significance threshold of 25% to identify potential predictors, which were then entered into a stepwise multiple linear regression model with a conventional significance level of 5%.

## Results

**Fig. 1:**
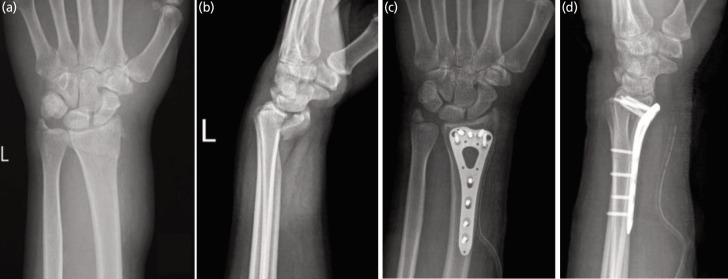
(a and b) Radiographs of 58-year-old female with an intra-articular radius fracture, (c and d) treated with volar locking plate fixation. Post-operative radiological measurements show radial height of 12.8mm, radial inclination of 19.6°, ulnar variance of – 1.9mm, and volar tilt of 5.6°.

A total of 78 patients were enrolled in the study comprising of 44 (56.4%) males and 34 (43.6%) females. Mean age of the patients was 50.3 + 14.7 (range 23-83) years. Majority of the patients had partial or complete intra-articular fractures. A case example is illustrated in Fig. 1 The patients’ demographic and fracture characteristics are delineated in Table I. The mean cumulative PRWE score was 42.4 + 6.6. The mean individual PRWE pain and PRWE function scores were 21.24 and 21.34, respectively. The mean post-operative radiological parameters are represented in Table I. Age demonstrated a strong positive correlation with PRWE scores, indicating poorer functional outcomes. A mild positive correlation was observed with palmar tilt, while radial height, radial inclination, and volar tilt showed modest negative correlations with PRWE scores, reflecting better outcomes (see Table II).

**Table I:** Patient demographics, fracture characteristics, and radiological parameters.

Demographics	
Age (years)	50.25 ± 14.7
Gender (Male/Female)	44 (56.4%) / 34 (43.6%)
Hand Dominance (Right/Left)	67 (85.9%) / 11 (14.1%)
Side of Injury (Right/Left)	28 (35.9%) / 50 (64.1%)
Radiographic Parameters	
Radial Height (mm)	12.11 ± 1.7
Radial Inclination (°)	20.33 ± 2.7
Palmar Tilt (°)	6.81 ± 3.4
Ulnar Variance (mm)	12.03 ± 1.1
Mode of Anaesthesia	
General Anaesthesia	71 (91%)
Regional Anaesthesia	7 (9%)
AO Classification	
A - Extra-articular	8 (10.3%)
B - Partial Intra-articular	37 (47.4%)
C - Intra-articular	33 (42.3%)
Functional Outcome	
PRWE Score	42.38 ± 6.6

**Table II:** Pearson's correlation coefficient for predictor variables.

Variable	Correlation co-efficient
Age	0.60
Radial height	-0.13
Radial Inclination	0.10
Palmar tilt	-0.12
Ulnar Variance	0.32

**Fig. 2:**
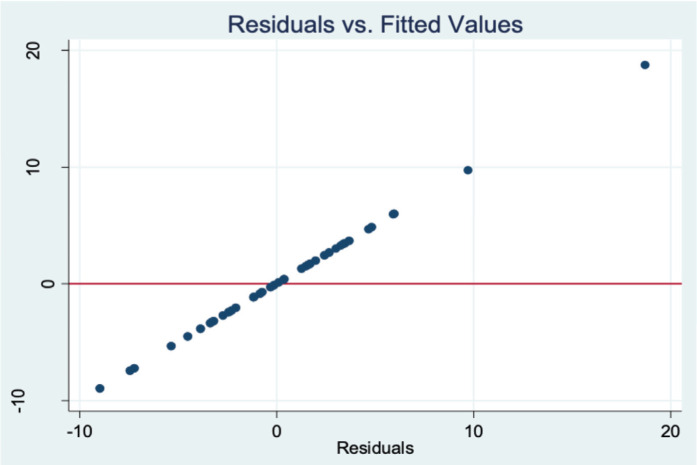
Scatter plot of residuals and fitted value from the final multiple linear regression model.

**Fig. 3:**
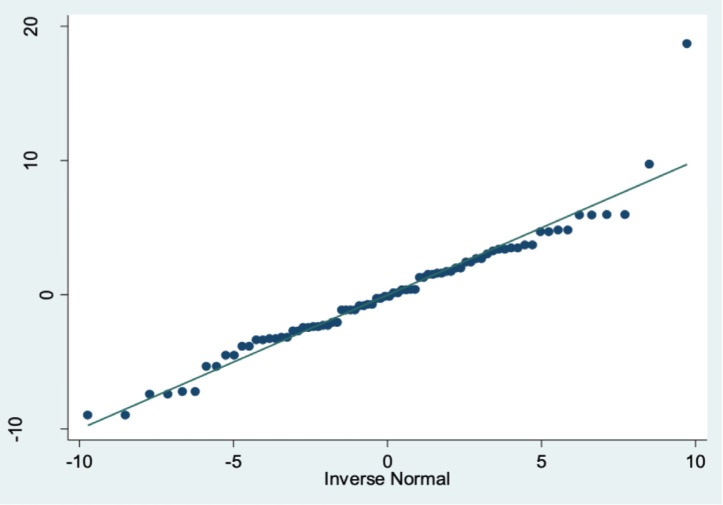
P-Q plot showing no evidence of heteroscedasticity.

**Fig. 4:**
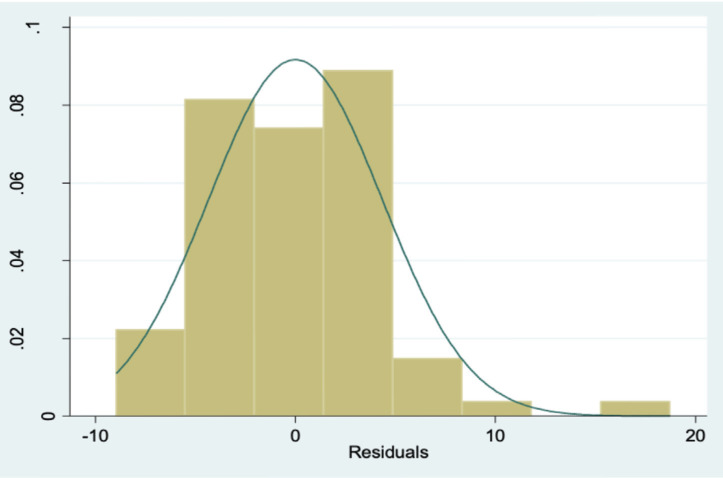
Histogram of residuals demonstrating a normal distribution.

**Fig. 5:**
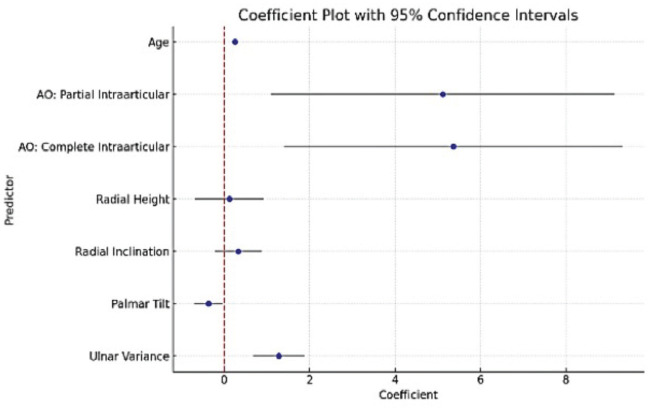
Co-efficient plot for predictor variables illustrating 95% confidence intervals

A step wise multiple linear regression analysis was done, and the final model included age, fracture type and the four radiological parameters of interest keeping cumulative level of significance at 5% (see Table III). Model adequacy and fit were checked after predicting fitted and residual values (see Fig. 2,  3, 4 and 5) and outliers were assessed. The final model coefficients are delineated in Table III. We observed that with each additional year of age, the outcome score increased by 0.26 units keeping all other predictor variables constant. This is statistically significant, suggesting age is positively associated with poorer functional outcomes (p=0.000). Similarly, increased ulnar variance is strongly associated with worse outcomes (coef. = 1.28; p<0.001).

**Table III:** Multiple linear regression model and regression co-efficient elaborating the association of the predictor variables and PRWE Score with 95% confidence interval.

Regression Co-efficient	Standard Error	Significance	95% CI	
Age	0.26	0.04	0.000	0.18 to 0.34
AO classification				
Partial Intra-articular	5.11	2.02	0.013	1.09 to 9.14
Intra-articular	5.36	1.98	0.009	1.41to 9.31
Radial Height	0.13	0.40	0.753	-0.68 to 0.93
Radial Inclination	0.33	0.28	0.232	-0.22 to 0.88
Palmar tilt	-0.36	0.17	0.035	-0.70 to -0.03
Ulnar variance	1.28	0.3	0.000	0.69 to 1.88
Constant (B0)	20.38	5.5	0.000	9.42 to 31.32

## Discussion

The findings of this study add novel evidence from a South Asian population, highlighting ulnar variance and age as stronger predictors of early functional outcomes than traditionally emphasised parameters such as radial inclination or height^13^. The findings of the current study will add to the increasing amount of data demonstrating the significance of anatomical reduction in fractures of the distal radius. The predictive value of radiological parameters in functional recovery has been the subject of conflicting findings in prior research. While some research shows a strong relationship between improved wrist flexion and maintained volar tilt, other research suggests that patient adherence to rehabilitation and soft tissue healing may have a greater influence on the results^14^. Although patient adherence to rehabilitation and soft tissue recovery are widely acknowledged as influential factors in functional outcomes, our study did not assess or control for compliance with physiotherapy protocols. Therefore, while we reference these aspects in contextualising our findings, they should be interpreted as potential—but unmeasured—modifiers of recovery rather than direct variables in our analysis.

In our cohort, the mean values for radial height (12.11 ± 1.7mm), radial inclination (20.33 ± 2.7°), palmar tilt (6.81 ± 3.4°), and ulnar variance (12.03 ± 1.1mm) showed notable deviations from established morphometric standards. In a study by Bilgin *et al*, conducted on the Turkish population, the average palmar tilt was 15.8 ± 4.4°, radial inclination 26.8 ± 3.6°, radial height 14.2 ± 2.1mm, and ulnar variance 0.7 ± 1.8mm^15^. Similarly, Mishra *et al* reported values in the Indian population with palmar tilt at 10.07 ± 5.28°, radial inclination 23.27 ± 7.42°, radial height 11.31 ± 4.9mm, and ulnar variance 0.66 ± 2.46mm^16^. In the Malaysian cohort studied by Chan *et al*, palmar tilt averaged 12.2 ± 3.18°, radial inclination 25.1 ± 3.35°, and ulnar variance −0.14 ± 1.24 mm^17^. Compared to these benchmarks, our cohort demonstrated reduced palmar tilt and radial inclination, and a markedly elevated ulnar variance, which may reflect residual deformity or population-specific anatomical variation following volar plate fixation. Unlike several earlier studies, our results suggest population-specific trends in anatomical alignment and functional recovery, reinforcing the importance of contextualised surgical planning.

This prospective cohort study examined the relationship between radiological alignment following volar locking plate fixation and short-term functional outcomes in patients with distal radius fractures. Our findings indicate that certain radiological parameters—specifically ulnar variance and palmar tilt along with age and fracture type, significantly influence recovery, as measured by the PRWE score at three months.

Age was found to be a strong predictor of poorer functional outcomes. With each additional year, PRWE scores increased significantly, indicating higher levels of pain and functional limitation (coef=0.26, CI: 0.18-0.34). This aligns with existing literature suggesting that older patients often have delayed or compromised recovery due to factors such as osteoporosis, comorbidities, and reduced tissue healing capacity^18,19^.

Increased ulnar variance—specifically shifting from a neutral to a more positive value—was the radiological parameter most strongly associated with poorer functional outcomes. In order to preserve distal radio-ulnar joint congruence and avoid changed load distribution across the wrist, it is biomechanically crucial to restore ulnar variance during fixation^9,20^. Palmar tilt also demonstrated a significant inverse relationship with PRWE scores, suggesting that inadequate restoration of this angle may impair wrist flexion and contribute to discomfort, corroborating earlier reports on its role in wrist mechanics^21^.

PRWE scores were also significantly related to fracture configuration. Patients with intra-articular fractures fared worse than those with extra-articular patterns, highlighting the complexity and severity of these injuries and the possibility of post-traumatic arthritis or incongruent joint surfaces even after surgical fixation. The mean differences in PRWE scores observed across age and ulnar variance groups exceed the minimally clinically important difference (MCID) threshold for PRWE, typically reported between 11.5 and 14 points, underscoring the clinical as well as statistical significance of our findings^22,23^. On the contrary, radial height and radial inclination in our last model revealed no notable correlations with PRWE scores but were included in the final model as these variables were part of the hypothesis. Though often used criteria for anatomical reduction, the influence of these variables on short-term functional results could be minimal if other elements, including ulnar variance and palmar tilt, are not properly handled^24^. Several previous studies have also found no consistent association between radiographic parameters and patient-reported functional outcomes following distal radius fracture treatment. For instance, Gutiérrez-Monclus *et al* observed that suboptimal fracture alignment—including residual dorsal angulation and radial shortening—did not significantly correlate with short- or medium-term recovery as assessed by validated tools such as PRWE, DASH, and VAS^25^. Similarly, Anzarut *et al* reported no meaningful differences in functional scores (including SF-12 PCS, SF-12 MCS, DASH, and patient satisfaction) between individuals who achieved acceptable radiographic reduction and those who did not^26^. These findings are in line with our study, where radial height and radial inclination demonstrated no significant correlation with PRWE scores, despite their theoretical relevance to wrist mechanics. This reinforces the possibility that soft tissue healing, pain perception, and rehabilitation may play more influential roles in determining recovery than radiological restoration alone, underscoring the need for further focused research to better define these relationships.

The strengths of the study are adequate sample size as compared to others like studies and extensive data handling and analysis; however, there are certain caveats. Complex intra-articular and severely comminuted fractures were not excluded to reflect the full clinical spectrum of distal radius injuries. However, this may introduce variability, and future studies should consider stratified analysis to assess the impact of fracture complexity on outcomes more precisely. While a three-month follow-up captures early recovery, it does not reflect long-term outcomes such as degenerative changes or chronic stiffness. This is acknowledged as a limitation, and future studies with extended follow-up are recommended. Potential confounding factors such as diabetes, osteoporosis, and adherence to physiotherapy protocols were not controlled in our study and may influence functional recovery. We recommend future investigations to include these variables in multivariate models. Though PRWE is a proven instrument, it is still a subjective measure, and future research could benefit from integrating objective functional assessments along with the subjective version of scores to better decipher this correlation.

## Conclusion

In this study, age, fracture type, ulnar variance, and palmar tilt were found to be significant predictors of functional recovery following volar locking plate fixation for distal radius fractures. Restoration of ulnar variance and palmar tilt appears especially important for achieving better short-term outcomes. These findings support the emphasis on precise radiological alignment during surgery and highlight the need to consider patient age and fracture complexity when counselling patients and planning treatment. Further research with longer follow-up is warranted to assess the long-term impact of these parameters on wrist function.
